# Polybenzimidazole dispersed polymer coated nanowires as efficient electrolytes for proton exchange membrane fuel cells

**DOI:** 10.1038/s41598-024-65955-9

**Published:** 2024-06-27

**Authors:** M. Abd Elkodous, Keiichiro Maegawa, Atsunori Matsuda

**Affiliations:** 1 Department of Electrical and Electronic Information Engineering, Toyohashi University of Technology, 1-1 Hibarigaoka, Tempaku-Cho, Toyohashi, Aichi 441-8580 Japan; 2 Next-Generation Energy Systems Group, Centre of Excellence ENSEMBLE3 Sp. z o.o., Wolczynska 133, 01-919 Warsaw, Poland

**Keywords:** PEM fuel cells, Polymer electrolytes, Nanowires, Proton conductivity, PBI, Membranes, Chemistry, Energy science and technology, Materials science

## Abstract

In this study, polymer-coated anisotropic inorganic nanowires dispersed in PBI matrix were introduced to construct 1D proton conducting channels within PBI. Ionic-liquid and solvothermal methods were used for the synthesis of ZrO_2_ and W_18_O_49_ NWs, which were coated with PVPA and PDDA polymers to increase their proton conductivity. Our results showed that, prepared membranes have amorphous nature due to the dominating presence of PBI. SEM analysis revealed the average thickness of membrane of about 36 µm. TG/DTA analysis detected lower weight loss of W_18_O_49_ NWs (total 2.8%) compared to ZrO_2_ NWs (18%). Proton conductivity analysis showed that, PDDA/W_18_O_49_ NWs possess relatively 4 times higher proton conductivity (4$\times$10^−4^ Scm^−1^) compared to PDDA/ZrO_2_ NWs (1$\times$10^−4^ Scm^−1^) at 80 ℃. In addition, PDDA-coated W_18_O_49_ NWs dispersed PBI membranes showed the highest fuel cell current density (1.2 A/cm^2^) and power density (215 mW/cm^2^) at 150 ℃ after 24 h which is nearly 2.5 times higher than pure PBI membrane. In addition, they exhibited the lowest in-situ proton resistance of about (0.47 Ω) compared with that of pure PBI membrane (0.8 Ω). Our results are introducing new concepts towards the development of thin and efficient polymer electrolyte membranes for PEM fuel cells.

## Introduction

The raised amounts of greenhouse gases in the atmosphere and the associated global warming and climate change, is pushing the world into carbon-neutrality^1^. The discovery of William R. Grove enabled the replacement of conventional fossil fuel engines with fuel cells that generate electricity from gaseous hydrogen (H_2_) and oxygen (O_2_)^2^. Since then, fuel cell technology has been developed into multiple types^3–5^. With respect to them, proton exchange membrane fuel cells (PEMFCs) possess promising characteristics such as high-power density, simplicity, quick start, pollutant-free emission, and it is suitable for portable power generation and small-scale electronic equipment^6^. Thus, PEMFCs are promising chemical-to-electrical energy conversion devices providing clean energy. However, the overall performance of fuel cells is not satisfactory, hampering their technological commercialization until now. In addition, there still many challenges and limitations need to be addressed such as the high cost of manufacture, due to the cost of electrode catalysts (precious metals), catalyst corrosion, and the poor performance of membrane electrode assembly (MEA) comprised of both electrodes (anode / cathode) and ion exchange membrane. The overall performance of PEMFCs is determined by their MEA. On the one hand, the electrodes (particularly the electrocatalysts) facilitate the electrochemical reactions that take place in the fuel cell. On the other hand, ion exchange membranes (ex: proton exchange, anion exchange, etc.) act as ions-conducting medium, fuel barrier, and electronic insulator^7^. Thus, development of highly efficient MEA can foster the ongoing efforts toward a commercialized fuel cell technology. The essential requirements for efficient electrocatalysts include activity to initiate the electrochemical reactions, selectivity with no undesired backward reactions, low poisoning resistance occurred with low purity fuels, and high stability / durability under different operating conditions^8,9^. In addition, high surface area to maximize active sites and thus the reaction rate, and high electrical conductivity. While, good proton exchange membranes (PEMs) should have good mechanical properties, low electronic conductivity, long-term stability, high protonic conductivity, and good stability in both reducing and oxidizing environments^10,11^.

Currently, carbon supported platinum-based electrodes (Pt/C) are widely employed in fuel cells due to their high oxygen reduction rate (ORR) and mass activity. While for the membrane, many kinds of polymers were investigated as the base matrix for PEMs including perfluorosulfonic acid-based membranes (ex: Nafion)^12^. Nafion has good protonic conductivity. However, it loses its conductivity at high temperatures affecting the power generation efficiency. Consequently, a humidifier is needed, implying a separate humidifier unit and control device, which increases the size and cost of the system. Otherwise, membrane structure gradually deteriorates, resulting in a significant increase in resistance. Furthermore, because perfluorinated ionomer (PFI) structurally contains fluorine, its production and disposal has became an environmental issue. Another example is polybenzimidazole (PBI)-based electrolytes, which requires no humidification^13^. In addition, it possesses outstanding properties such as good chemical and thermal stabilities, excellent film-forming properties, superior toughness, promising mechanical characteristics and sufficient gas impermeability. However, pure PBI polymers have poor proton conductivity, making the doping process with strong acid (ex: phosphoric acid (PA)) inevitable. By using PA-doped PBI electrolytes with high molar ratio against PBI unit (Phosphoric acid doping level: PADL about 10–15), it is possible to make non-humidified PEMFCs. However, PA-doping leads to membrane strength deterioration due to the excessive hydrophilicity and PA leaching resulting in Pt dissolution and carbon corrosion. Recently, the effect of 1D and / or 2D anisotropic materials for capturing PA and forming continuous PA channels for improving proton conductivity without increasing PA doping level was reported^7^.

In this study, we attempted to introduce anisotropic inorganic materials with enhanced PA affinity utilizing anionic/cationic polymer coating to maintain the proton conductivity of PA-PBI with reduced PADL of 8, by incorporating a variety of solid proton conductors to form composite PBI membranes^14^. Specifically, two kinds of inorganic nanowires (NWs), ZrO_2_ NWs and W_18_O_49_ NWs as inorganic fillers coated with poly (vinylphosphonic acid, PVPA) ionic polymer and poly (diallyldimethylammonium chloride, PDDA) cationic polymer were used. Basically, PA acts as an anion in the membrane and can electrically interact with the NWs or the polymer coated on the NWs, forming an efficient 1D proton channel on the surface. It is expected that different inorganic wire/polymer combinations will affect not only the PA affinity in the film, but also the dispersibility, morphological properties, and even mechanical properties of the membrane. Therefore, we performed a multifaceted evaluation of the fillers prepared in each combination and the PA-PBI electrolyte membranes to which they were added and investigated their effects on the performance of non-humidified PEMFC.

## Materials & methods

### Materials

Zirconium(IV) propoxide solution 70 wt.% in 1-propanol (Zr(OPr^n^)_4,_ Sigma, Japan), ethylene glycol (EG, Sigma, Japan),1-butyl-3-methylimidazolium tetrafluoroborate (BMImBF_4_, Sigma, Japan), absolute ethanol (EtOH, Sigma, Japan), tungsten(VI) chloride (WCl_6_, Sigma, Japan), PVPA solution 30 wt.% (Sigma, Japan), PDDA solution 20 wt.% (Sigma, Japan), N,N-dimethylacetamide (DMAc, Wako, Japan), polybenzimidazole solution 10 wt.% in DMAc, Mw ∼ 26000 (PBI, Sato Light Industrial, Japan), phosphoric acid 85% (PA, Wako, Japan), and 0.5 mg cm^−2^ Pt-loaded carbon paper (Pt/C, Fuel Cell Earth, CTM-GDE-01, USA). All materials and reagents were used as received without further processing.

### Methods

#### Preparation of Zirconium oxide nanowires (ZrO_2_ NWs)

ZrO_2_ NWs were prepared using an ionic-liquid route as previously reported^15^. Firstly, (3.36 g) of Zr(OPr^n^)_4_ was added to (60 mL) EG under magnetic stirring under ambient conditions. Then (4 g) BMImBF_4_ was added to the above solution. After that, the formed mixture was transferred into a 100 mL Teflon-lined autoclave which was heated at 160 ℃ for 46 h. After cooling down to room temperature naturally, the formed white precipitate was collected and washed 5 times by EtOH via centrifugation, then dried under vacuum at 60 ℃. Finally, dried powder was heat-treated at 500 ℃ for 2 h.

#### Preparation of Tungsten oxide nanowires (W_18_O_49_ NWs)

W_18_O_49_ NWs were prepared by a solvothermal method in literature^16^ with slight modifications. (500 mg) of WCl_6_ was dissolved in (60 mL) EtOH until clear yellow solution is obtained. Then, the formed solution was inserted into a 100 mL Teflon-lined autoclave heated at 180 ℃ for 24 h. After natural cooling, the formed blue powder was collected, washed 4 times by EtOH by centrifugation, and dried at 60 ℃ in a vacuum oven.

#### Surface modification of the prepared NWs

Proton conductivity of the prepared NWs was improved through PVPA and PDDA surface coating as the following: NWs of both types were dispersed in (20 mL) distilled water (D.W.) using sonication for 1 min. Then, PVPA and PDDA polymers were added to the NWs’ dispersion (polymer ratios were 20 wt.%, 50 wt.%, and 65 wt.% compared with the NWs). After that, the mixtures were left under continues stirring overnight. Finally, the coated NWs were collected, washed 1 time by D.W. and dried under vacuum at 60 ℃.

#### Preparation of polymer-coated NWs’ dispersed PBI membranes

Many polymer-coated NWs’ dispersed PBI membranes (polymer-coated NWs = 2 wt.%) were prepared via a simple casting method developed by Maegawa et al., as shown in Fig. S.1 ref^17,18^. First, (9.62 mg) of NWs was dissolved in (18.81 g) DMAc using ultrasonication for 1 h. Then, (4.71 g) of PBI was added to the above solution and left under magnetic stirring for another 1 h. After that, sonication took place for 30 min to form a clear and homogonous solution. Then, final solution was casted in petri dishes (diameter = 97 mm) and heat-treated for 24 h using a programmed gradual heat increase from 60 to 120 ℃. Finally, obtained membranes were treated with hot water for 5 h at 90 ℃ to remove any residual DMAc. As a reference, pristine PBI membrane (with no NWs) was prepared by the same method for comparison. 4 membranes are presented in this study named, W_18_O_49_/PBI, PVPA/W_18_O_49_/PBI, PDDA/W_18_O_49_/PBI, and Pure PBI membrane. The thickness of the prepared membranes was in the range of 35 to 55 µm.

#### Phosphoric acid (PA) doping of membranes

Before fuel cell measurements, membranes (2 cm × 2 cm) were doped with PA doping level of 8 mol, (PADL = 8) by immersion in PA, then membranes were stretched between two glass slides at 60 ℃ for 1 h, PADL was calculated using Eq. (1):

1 $$ {\text{PADL}} = \left( {\frac{{{\text{W}}_{{{\text{doped}}}} - {\text{W}}_{0} }}{{{\text{W}}_{0} }}} \right){ } \times \left( {\frac{{{\text{M}}_{{{\text{w}}t \left( {PBI} \right)}} }}{{{\text{W}}_{{{\text{w}}t \left( {PA} \right)}} }}} \right) \times 100{ ,} $$

where W_o_ and W_doped_ are the weight of membranes before and after PA doping. While M_wt_ represents the molecular weight.

#### PA leaching analysis

To analyze PA retention by the membranes, PA leaching test was carried out according to our previous study^19^. Membranes with PADL = 8 were exposed to steam (from ion exchange water at 100 ℃) and after specific time intervals, the remaining amount of PA (after gentle wiping) was calculated by measuring the weight loss over time.

#### Swelling ratio & water uptake test

The manufactured membranes were placed into 85% PA solution. Subsequently, swelling ratio and water uptake percentage were assessed according to Eqs. (2, 3), respectively. Prior to the immersion in PA, the initial weight (m_o_), thickness (T_o_), and area (A_o_) of the membranes were recorded. Following PA doping at 60 ℃, the weight (m_doped_), thickness (T_doped_), and area (A_doped_) were determined following a careful wipe (to remove excess PA solution). The PA-doped membranes were then dried in an oven at 100 ℃ for 1 h, and the mass (m_dry_) was measured.

2 $$ {\text{Swelling ratio}} = \frac{{{\text{A}}_{{{\text{doped}}}} {\text{T}}_{{{\text{doped}}}} - {\text{A}}_{0} {\text{T}}_{0} }}{{{\text{A}}_{0} {\text{T}}_{0} }} \times 100{ ,} $$

3 $$ {\text{Water uptake}} = \frac{{{\text{m}}_{{{\text{doped}}}} - {\text{m}}_{{{\text{dry}}}} }}{{{\text{m}}_{0} }} \times 100. $$

#### Membrane-electrode assembly (MEA) and evaluation of fuel cell performance

1 × 1 cm^2^ and 1.5 × 1.5 cm^2^ Pt/C electrocatalyst sheets were utilized as the anode and cathode, respectively. To evaluate fuel cell performance, PA-doped membranes (PADL = 8) were sandwiched between the two electrodes to fabricate MEAs. Then fuel cell testing system (Auto PEM system, Toyo Technica, Japan) supplying H_2_ and O_2_ at a flow rate of 100 mL min^−1^ to the anode and cathode, respectively was employed, experiments were performed at 150 ℃ (anhydrous conditions).

#### Characterization of the prepared samples

Phase and crystallinity were examined by X-ray diffraction (XRD) analysis performed with a (Rigaku, Ultima IV X-ray diffractometer, Japan, utilizing Cu-Kα radiation (λ = 1.54 Å) and operating at 30 mA – 40 kV. Scanning electron microscopy (SEM) conducted with a (HITACHI S-4800, Japan), accompanied by an energy-dispersive X-ray spectroscopy (EDX) unit (Oxford Instruments, AZtecLive Lite, Ultim Max 40), was employed to determine the morphology, average length, and diameter of NWs. The surface charge of NWs (Zeta potential) at various pH levels was investigated using an (ELS-Z1NT analyzer, Photo OTSUKA ELECTRONICS, Japan). To assess the mechanical strength of the membranes, a Tensilon apparatus (RTF-1250, Japan) was used, with a tensile test speed of 1 mm min^−1^. X-ray photoelectron spectroscopy (XPS) was carried out on a (PHI Quantera SXM Scanning X-ray Microprobe, ULVAC-Phi, Inc., Japan), for analyzing elemental composition and chemical states. Thermal stabilities of the NWs were assessed in air through TG/DTA analysis conducted on (Rigaku, Thermo plus EVO2, Japan) with heating rate of 10 ℃ min^−1^. Lastly, temperature-dependent proton conductivity measurements were carried out via AC impedance spectroscopy over a frequency range of 1 Hz-10 MHz using (Solartron, SI 1260) under dry N_2_ conditions.

## Results & discussion

### Characterization of the prepared NWs and membranes

**Figure 1:**
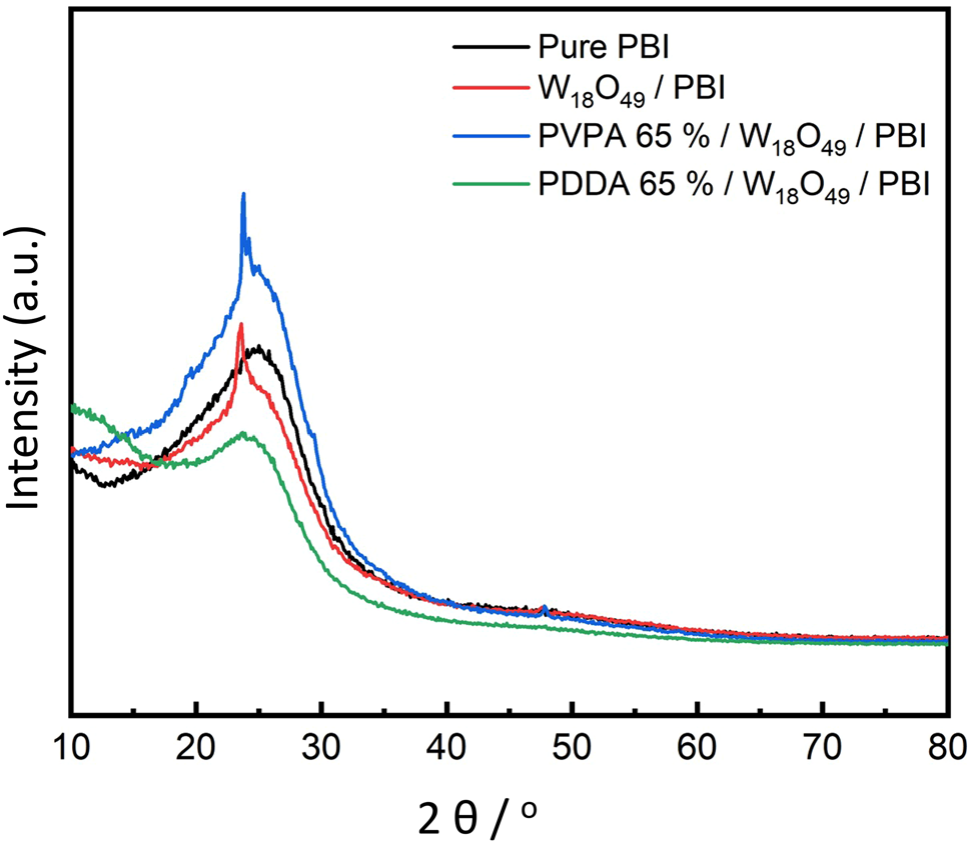
XRD analysis of the prepared membranes.

XRD pattern of the prepared membranes is presented in Fig. 1. As clearly observed, for pure PBI membrane, a broad peak is detected at 2θ = 25^°^, which is reflecting its amorphous nature. While for W_18_O_49_ NWs’ dispersed membranes, a slight shift towards the smaller angle is recorded, which is attributed to the change in the internal structure and / or disruption of the ordered stacking of PBI^20,21^. Fig. S.2a,b shows the XRD patterns of bare ZrO_2_ and W_18_O_49_ NWs. Firstly, for W_18_O_49_ NWs, many peaks are recorded at 2θ = 14.5^°^, 22.5^°^, 26.3^°^, 36.2^°^, 50.1^°^, 54.4^°^, and 55.2^°^ corresponding to the crystallographic planes (301), (010), (203), (114), (020), (315), and (523). The recorded peaks are in good agreement with those recorded for the monoclinic phase of Tungsten oxide (JCPDS No. 712450)^22^. It is worth mentioned that unassigned peaks are related to other metal oxide forms of Tungsten oxide (ex. WO_3_)^23^. Secondly for ZrO_2_ NWs, after heat treatment at 500 ℃ for 2 h, recorded peaks at 2θ = 24.6^°^ (110), 28.8^°^(− 110), 32.3^°^(111), 34.6^°^(020), 49.3^°^(− 202), and 50.2^°^(022) which well-matched with the monoclinic phase of ZrO_2_ (JCPDS No. 37–1484)^24^, however before heat treatment, the formed phase of the corresponding peaks is unknown^15^.

**Figure 2:**
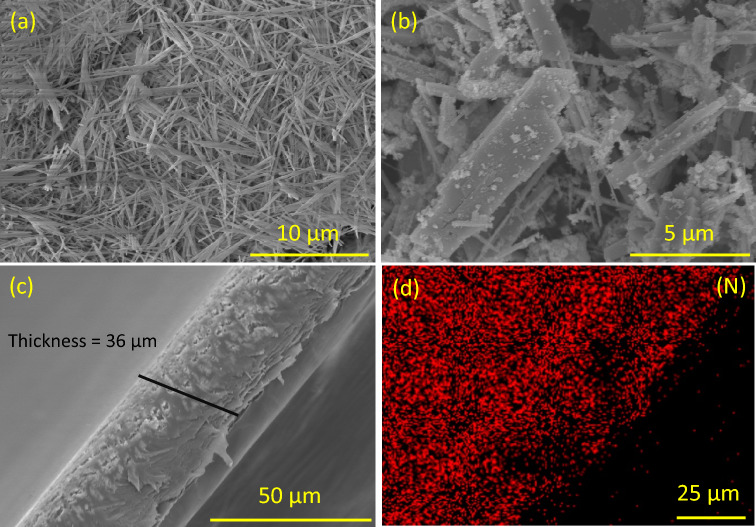
SEM analysis before heat treatment of (**a**) bare ZrO_2_ NWs (**b**) bare W_18_O_49_ NWs, (**c**) Cross-sectional analysis of W_18_O_49_/ PBI membrane, and (**d**) its EDX mapping for N_2_ element.

SEM / EDX analysis of the prepared NWs and membranes is shown in Fig 2a–d. ZrO_2_ NWs are thin and elongated with an average length of about 20 µm and diameter of 50 nm (a). While W_18_O_49_ NWs are shorter, thick, and agglomerated in bundles Fig. 2b**.** The cross-sectional image of W_18_O_49_/ PBI membrane Fig. 2c reveals a membrane thickness of about 36 µm. Finally, Fig. 2d shows the EDX mapping of N element corresponding to the PBI membrane. After heat treatment, the structure of ZrO_2_ NWs is significantly changed, and agglomeration occurs as revealed in Fig. S.3a**.**

**Figure 3:**
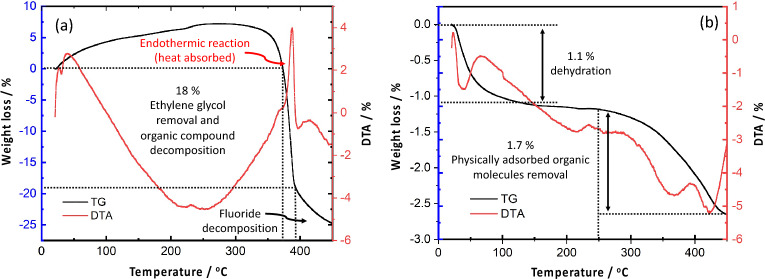
TG/DTA analysis of (**a**) bare ZrO_2_ NWs and (**b**) bare W_18_O_49_ NWs.

Thus, thermal stabilities of the bare NWs were evaluated using TG/DTA analysis from room temperature up to 450 ℃ as presented in Fig. 3a,b, the associated weight loss of W_18_O_49_ NWs was light (total 2.8%) compared to ZrO_2_ NWs (18%), two main regions of weight loss for W_18_O_49_ NWs were detected, the first one (1.1%) due to dehydration (from room temperature to up to 250 ℃), and another 1.7% weight loss because of the removal of physically adsorbed organic molecules (from 250 to 450 ℃). While the removal of ethylene glycol resulted in almost (18%) weight loss in case of ZrO_2_ NWs.

To estimate the net surface charge of the NWs affecting the affinity with coating polymers which in turns affect PA interaction and homogeneity in the membranes, Zeta potential/surface charge analysis of bare ZrO_2_ NWs, W_18_O_49_ NWs, and after their PVPA coating is carried out and results are summarized in Table 1. In the acidic medium (actual fuel cell working environment), W_18_O_49_ NWs possessed net negative charge (Zeta potential = − 26.96). On the contrary, ZrO_2_ NWs had net positive charge (Zeta potential = + 24.35). Thus, selecting the proper polymer for NWs’ coating is important to facilitate the electrical PA affinity and creating continuous 1D H^+^ conducting channels on NWs, as well as to fabricate homogeneously dispersed membranes. Taking into account that, PVPA possesses anionic charge while PDDA exhibits cationic charge, the suitable polymer/NWs combinations can be expected as follows: ZrO_2_/PVPA and W_18_O_49_/PDDA. PVPA (having protonic acid groups) is expected to have high proton conductivity itself. On the other hand, PDDA has the potential to electrically interact with anionic PA when it is incorporated in the PA-doped membranes resulting in an effective proton conductive channel, although PDDA itself has negligible proton conductivity.

**Table 1:** Zeta potential and net surface charge of the prepared ZrO_2_ NWs and W_18_O_49_ NWs at different pH.

	ZrO_2_ NWs	W_18_O_49_ NWs	PVPA coated ZrO_2_ NWs	PVPA coated W_18_O_49_ NWs
pH 3	+ 24.35	− 26.96	− 2.54	− 27.46
pH 7	+ 6.34	− 48.05	− 35.51	− 54.55
pH 11	− 54.70	− 49.31	− 48.64	− 64.17

**Figure 4:**
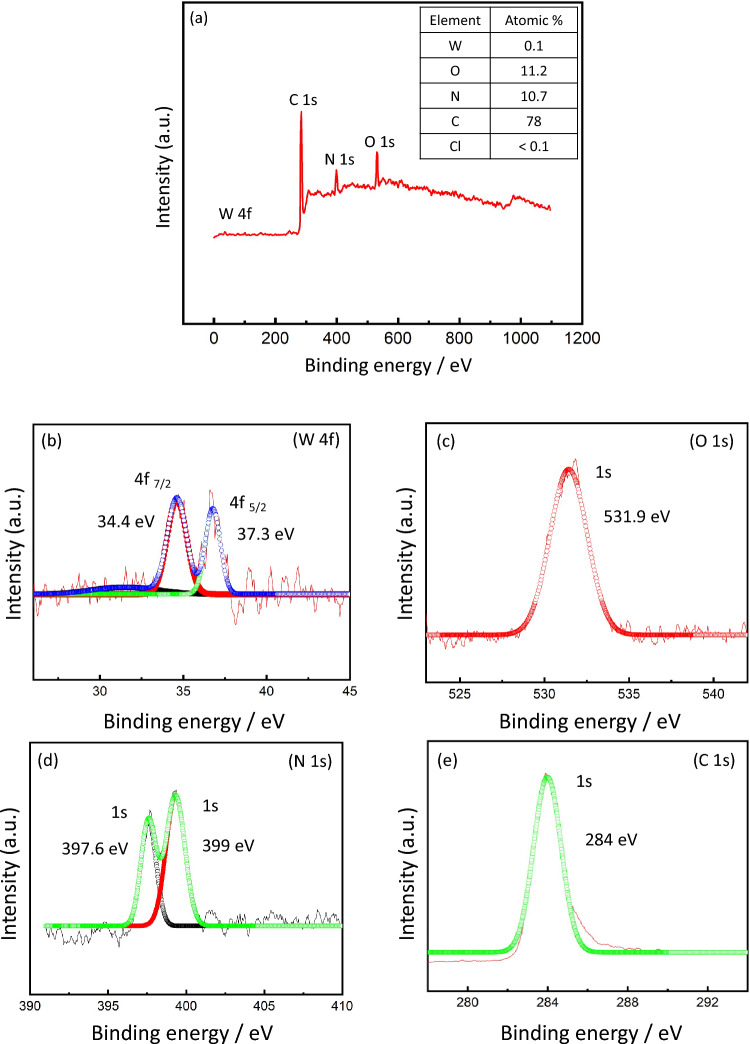
XPS analysis of PDDA/ W_18_O_49_/PBI membrane showing the atomic % of the main elements, (**a**) survey analysis, and deconvoluted spectra of (**b**) W 4f., (**c**) O 1s, (**d**) N 1s, and (**e**) O 1s.

Figure 4 shows the XPS analysis of the prepared PDDA/W_18_O_49_/PBI membrane. Figure 4a shows the survey study, where many peaks associated with all substantial elements are observed along with their atomic percentages. While the deconvoluted peaks of the main elements (W, O, N, and C) are shown in Fig. 4b–e. For W 4f., two main peaks at (34.4 and 37.3 eV) are detected, corresponding to (4f. _7/2_ and 4f. _5/2_), respectively, confirming the presence of W^+6^ state^25^. While for N 1s, the two peaks recorded at (397.6 and 399 eV) are due to the W–N bond configuration. For O1s, the characteristic peak of oxygen in metal oxides was observed at (531.9 eV) and confirming the presence of hydroxyl groups. Finally, for C1s, one main peak at (284 eV) was detected corresponding to C = C bond^26^.

### Proton conductivity of the prepared polymer-coated NWs

**Figure 5:**
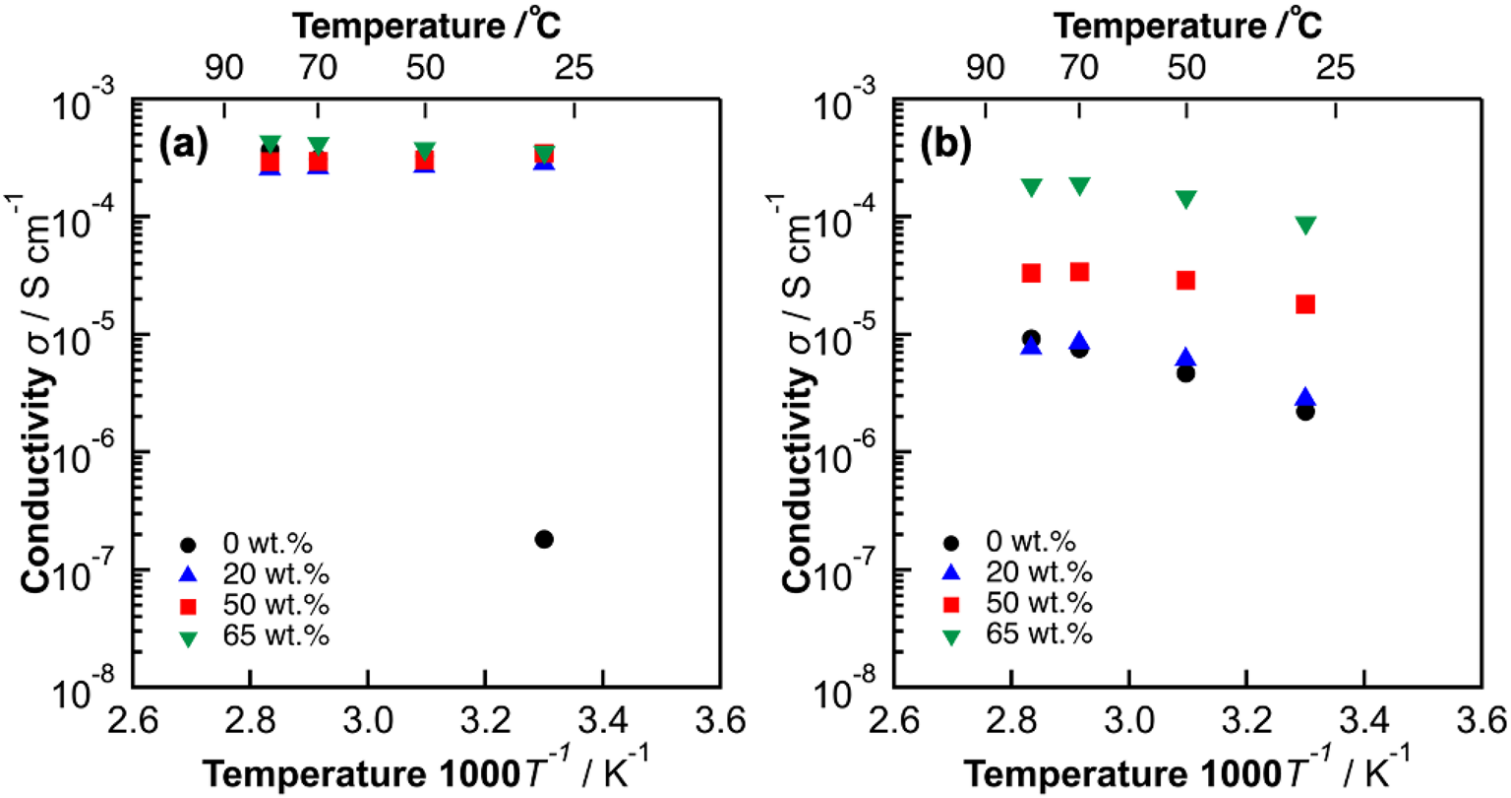
Proton conductivity of (**a**) PDDA coated W_18_O_49_ NWs (powder) and (**b**) PDDA coated ZrO_2_ NWs (powder) at 80% relative humidity.

To confirm the effect of polymer coating of NWs on proton conductivity, a set of proton conductivity measurements at a relative humidity of 80% were carried out. Figure 5a,b shows proton conductivity of PDDA/W_18_O_49_ and PDDA/ZrO_2_ NWs, it can be seen that, by increasing the polymer percentage (from 20 to 65 wt.%) and temperature (from 30 to 80 ℃), conductivity increases, and PDDA/W_18_O_49_ NWs possess relatively 4 times higher proton conductivity (4$\times$10^−4^ Scm^−1^) compared to PDDA/ZrO_2_ NWs (1$\times$10^−4^ Scm^−1^) at 80 ℃. In addition, proton conductivity comparison of PVPA/ W_18_O_49_ and PDDA/ W_18_O_49_ NWs is shown in Fig. S.4. Although both W_18_O_49_ NWs and PVPA have same charge (negative) the proton conductivity of W_18_O_49_ NWs increased upon coating with PVPA polymer, this can be attributed to the phosphoric acid groups of PVPA which possess good proton conductivity compared with the OH groups of PDDA polymer, overpassing the electrostatic repulsion which might occur over the surface of W_18_O_49_ NWs. According to the results mentioned above, W_18_O_49_-based NWs exhibited slightly better proton conductivity than ZrO_2_-based NWs. In addition, PVPA coated W_18_O_49_ NWs showed higher performance derived by the acidic groups, with respect to PDDA coated W_18_O_49_ NWs.

### Fuel cell performance analysis

**Figure 6:**
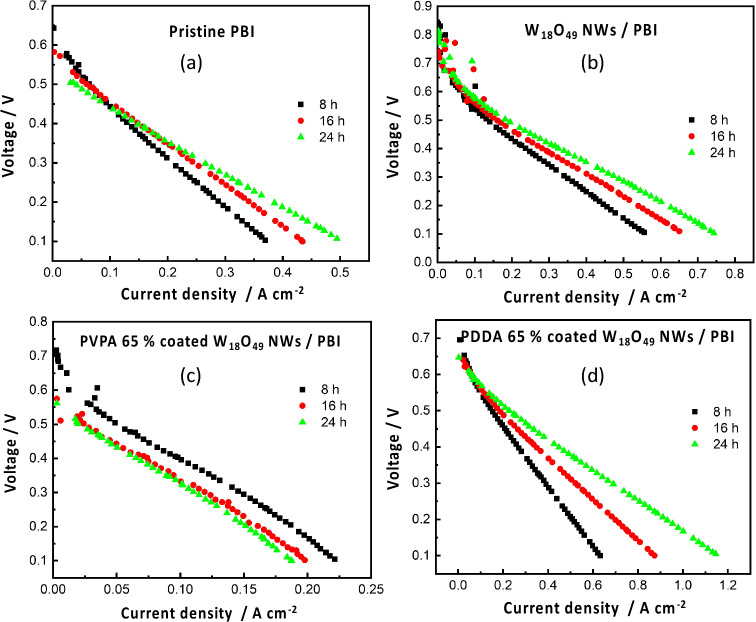
C-V curves of the prepared (**a**) Pure PBI membrane, (**b**) W_18_O_49_/PBI membrane, (**c**) PVPA 65%/ W_18_O_49_/PBI membrane, and (**d**) PDDA 65%/ W_18_O_49_/PBI membrane.

**Figure 7:**
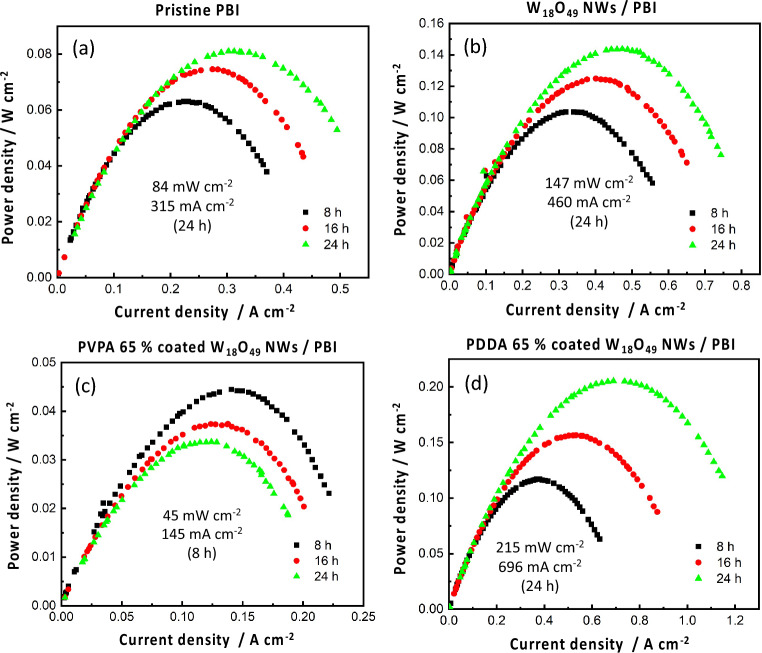
Power density curves of the prepared (**a**) Pure PBI membrane, (**b**) W_18_O_49_/PBI membrane, (**c**) PVPA 65%/ W_18_O_49_/PBI membrane, and (**d**) PDDA 65%/ W_18_O_49_/PBI membrane.

Figure 6a–d presents the current–voltage curves of the prepared membranes, while power density curves are shown in Fig. 7a–d. PDDA/W_18_O_49_/ PBI membrane showed the maximum current density of 1.2 A/cm^2^ Fig. 6d and its open circuit voltage (OCV) (at current density = 0 A/cm^2^) was maintained for 24 h. In addition, it showed the maximum power density of 215 mW/cm^2^ after 24 h which is 2.5 times higher than pure PBI membrane Fig. 7d. This result can be attributed to an improved protonic conductivity. It is worth to mention that OCV was slightly different for the prepared membranes, with a maximum value of about (0.85 V after 8 h) exhibited by W_18_O_49_/ PBI membrane Fig. 6b, while a minimum value of (0.65 V) was recorded for pure PBI membrane Fig. 6a. The decreasing of OCV over time is attributed to the gas leakage between the anode and cathode electrodes due to membrane penetration^27^. Thus, the polymer coated NWs may bring morphological and mechanical improvements, resulting in a reduced gas permeability. Another observation is related to the slope of the linear part of the I-V curve associated with membrane resistance, PDDA/W_18_O_49_/ PBI membrane exhibited the lowest resistance (slope) value of about (0.47 Ω) followed with W_18_O_49_/ PBI membrane (0.63 Ω), this smaller slope compared with that of pure PBI membrane (0.8 Ω), further confirming the improved protonic conductivity with respect to pure PBI membrane due to the absence of polymer-coated NWs. The dominant reason for this improvement is the PA coordination on the PDDA/W_18_O_49_ surface derived by the positively charged PDDA, forming 1D proton conducting channels on an anisotropic wire surface.

**Figure 8:**
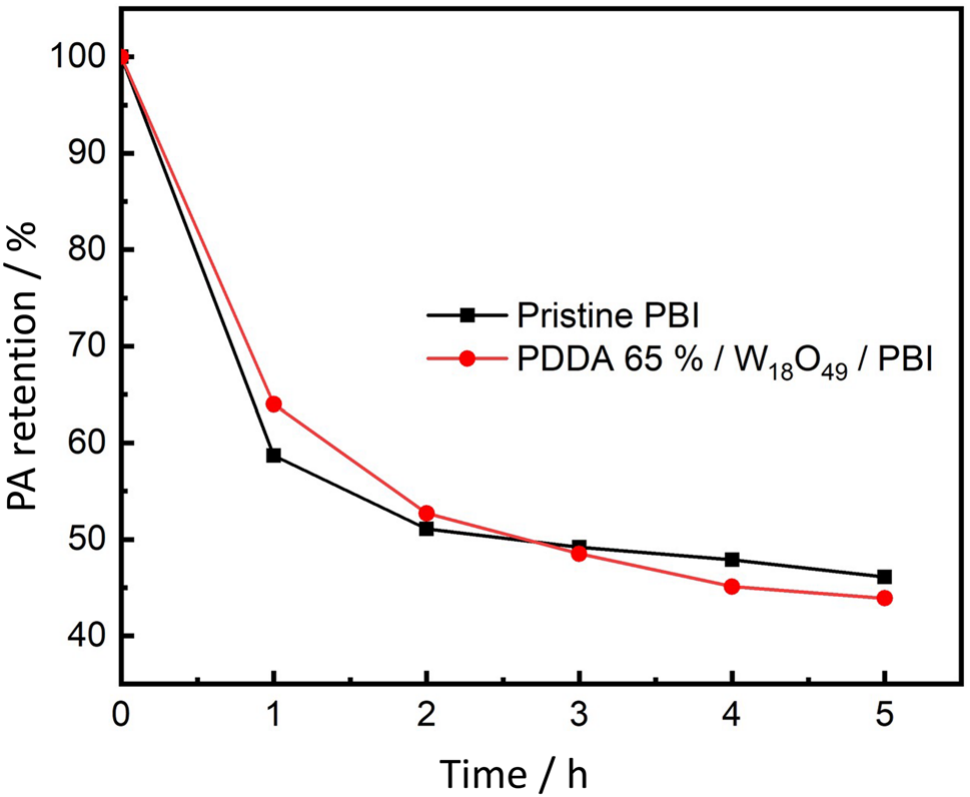
PA leaching test of Pure PBI and PDDA 65%/ W_18_O_49_/PBI membranes.

The OCV dramatically decreased after 24 h in case of pure PBI and PVPA/W_18_O_49_/ PBI membrane Fig. 6c. This significant decrease in OCV is due to either deterioration of catalyst performance over time due to corrosion or infiltration caused by PA leaching which results in an inadequate reaction at the three-phase interface^28^ or H_2_/O_2_ gas permeation due to membrane rupture and / or cracks. Thus, it can be concluded that, NWs’ dispersion in PBI membranes led to both PA retention as confirmed by Fig. 8, maintenance of proton conductivity pathway over time, and the suppression of membrane cracks by providing mechanical strength to the membranes as revealed by Fig. S.5. Another reason can be the homogeneity of membranes and suppression of NWs’ agglomeration maintained by their proper surface modification as shown in Fig. S.6, PDDA possesses net positive charge which favors the formation of a smooth layer over the surface of W_18_O_49_ NWs having net negative charge, while PVPA and W_18_O_49_ NWs repulsive forces cause the agglomeration of PVPA-coated W_18_O_49_ NWs in PBI matrix. These results indicate that the PDDA had multiple advantages as a coating polymer, i.e., improved homogeneity resulting in the fuel cell stability and high proton conductivity due to the PA affinity.

**Figure 9:**
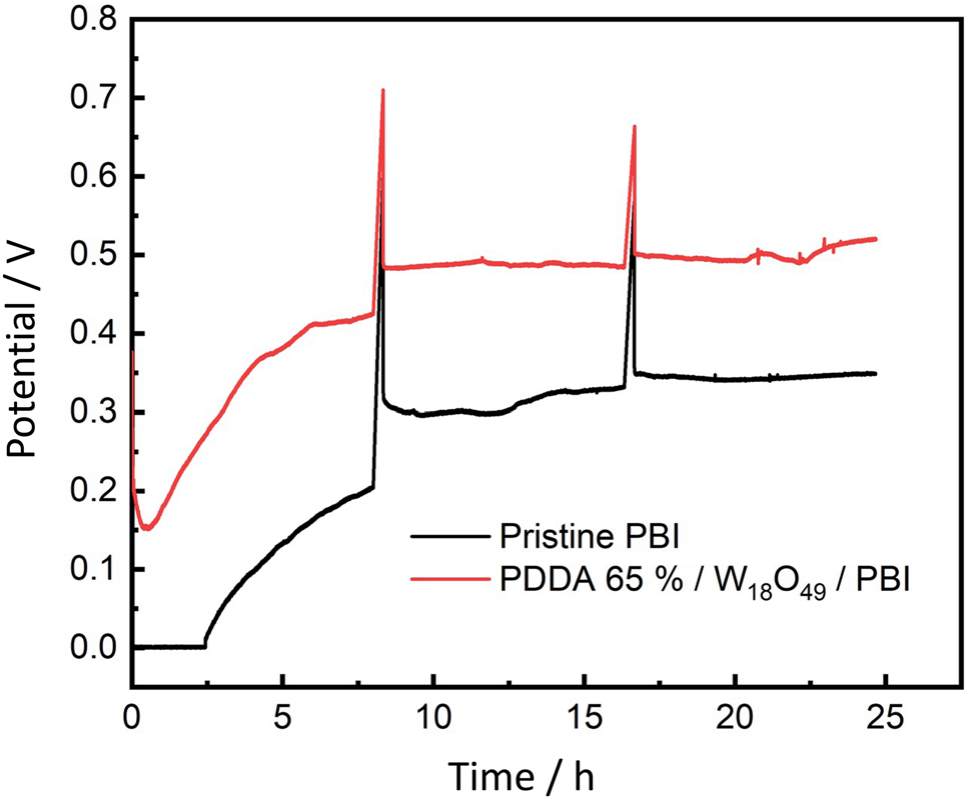
Potential over time at a constant current density of 0.2 Acm^−2^ for 24.

The constant current stability performance of PDDA/W_18_O_49_/ PBI and pure PBI membranes with a constant current density of 0.2 Acm^−2^ for 24 h is depicted in Fig. 9. The recorded potential of PDDA/W_18_O_49_/ PBI membrane tend to increase over time, this behavior can be related to PA leaching from PBI membrane and PA infiltrating into PBI electrode interface as seen in Fig. 8, up to 3 h, PA retention of PDDA/W_18_O_49_/ PBI membrane was better than that of pure PBI membrane, however with time, PA tend to leach from the membrane causing activation of the catalyst and increasing of the protonic conductivity and the associated electrochemical reactions at the 3-phase boundary leading to elevated potential value over time. This behavior is typical in PEM fuel cells^29,30^.

**Figure 10:**
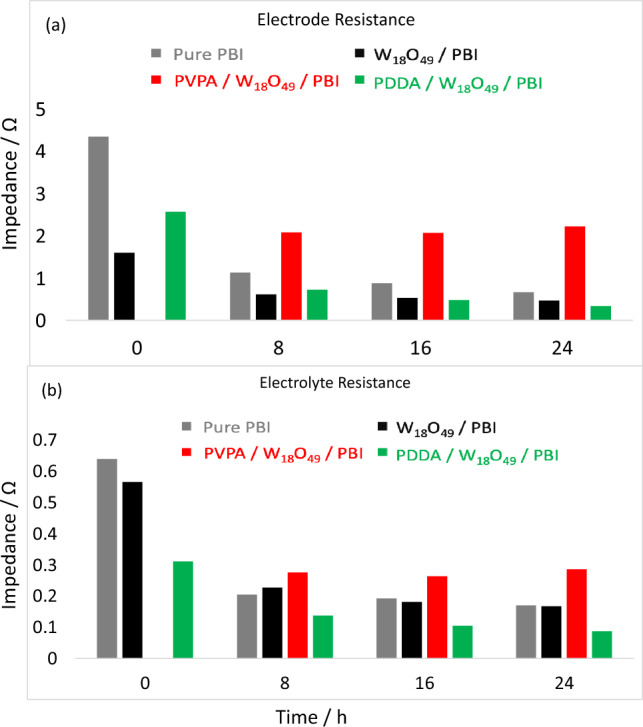
EIS analysis showing (**a**) electrode residence and (**b**) electrolyte (PEM) resistance of the prepared samples.

Figure 10a,b shows the calculated electrode and electrolyte resistance from electrochemical impedance spectroscopy analysis of the prepared membranes. It is obvious that, at 24 h, PDDA/W_18_O_49_/ PBI membrane possessed the lowest electrode and electrolyte (membrane) resistance which is favorable for high current and power densities. It is worth to mention that, values of electrolyte resistance in Fig. 10b are smaller than those shown in Fig. 6, that’s because in Fig. 6, values are corresponding to both electrolyte and interface resistances, while in Fig. 10b are solely due to the electrolyte resistance^31^.

## Conclusion

ZrO_2_ and W_18_O_49_ NWs were firstly prepared by ionic-liquid and solvothermal methods and then coated with PVPA and PDDA polymers to reduce the required amount of PA doping in the PBI membrane through increasing their protonic conductivity. Then, the prepared nanowires (2 wt.%) were dispersed in PBI matrix to form 4 polymer electrolyte membranes for PEM fuel cells.

Our results showed the importance of surface charge in fabricating membranes with uniform dispersion of nanowires. In addition, mechanical strength analysis revealed that, nanowires’ dispersion in PBI matrix increased the tensile strength of the formed membrane with respect to pure PBI membranes. PDDA/W_18_O_49_/ PBI membrane showed the maximum current and powder densities of 1.2 A/cm^2^ and 215 mW/cm^2^ after 24 h which is 2.5 times higher than pure PBI membrane. The improved fuel cell performance of PDDA/W_18_O_49_/PBI membrane than that of PVPA/W_18_O_49_/PBI, is attributed to the construction of proton conductive pathway because of the increased PA affinity, and the suppression of membrane mechanical degradation (leading to membrane cracks, gas crossover, and lower OCV). The advantages of the introduction of anisotropic nanowires in membrane thinning, mechanical properties improvement, surface charge role in membrane uniformity, and type of coating polymer for high PA affinity were revealed.

### Supplementary Information

Supplementary Information.

## Data Availability

Data are provided within the manuscript and / or supplementary information files.
