# Supplementary material for: Polybenzimidazole dispersed polymer coated nanowires as efficient electrolytes for proton exchange membrane fuel cells
Source: Sci Rep. 2024 Jun 27;14:14884. doi: 10.1038/s41598-024-65955-9 (PMC11211458; doi:10.1038/s41598-024-65955-9)
Supplement: Supplementary file 1 — Supplementary Information. [file 41598_2024_65955_MOESM1_ESM.docx]

**Polybenzimidazole Dispersed Polymer Coated Nanowires as Efficient Electrolytes for Proton Exchange Membrane Fuel Cells**

**M. Abd Elkodous^1,2^, Keiichiro Maegawa^1,3^, Atsunori Matsuda^1, *^**

^1,^ Department of Electrical and Electronic Information Engineering, Toyohashi University of Technology, 1-1 Hibarigaoka, Tempaku-cho, Toyohashi, Aichi 441-8580, Japan.

^2,^ Center for Nanotechnology (CNT), School of Engineering and Applied Sciences, Nile University, Sheikh Zayed, Giza16453, Egypt

^3,^ Next-Generation Energy Systems group, Centre of Excellence ENSEMBLE3 sp. z o.o., Wolczynska 133, Warsaw, 01-919, Poland.

***Corresponding author**

**1- Prof. Atsunori Matsuda**, Department of Electrical and Electronic Information Engineering, Toyohashi University of Technology, 1-1 Hibarigaoka, Tempaku-cho, Toyohashi, Aichi 441-8580, Japan, Email: [matsuda.atsunori.hh@tut.jp](mailto:matsuda.atsunori.hh@tut.jp).

**Fig. S.1:** Step-by-step preparation of polymer-coated NWs’ dispersed PBI membranes (polymer-coated NWs = 2 wt.%), prepared via a simple casting method.

**Fig. S.2:** XRD analysis of a) bare ZrO_2_ NWs (before and after heat treatment) and b) bare W_18_O_49_ NWS.

**Fig. S.3:** a) SEM analysis of ZrO_2_ NWs after heat treatment (at 500^o^C for 2h) and b-d) EDX mapping of W_18_O_49_/PBI membrane.


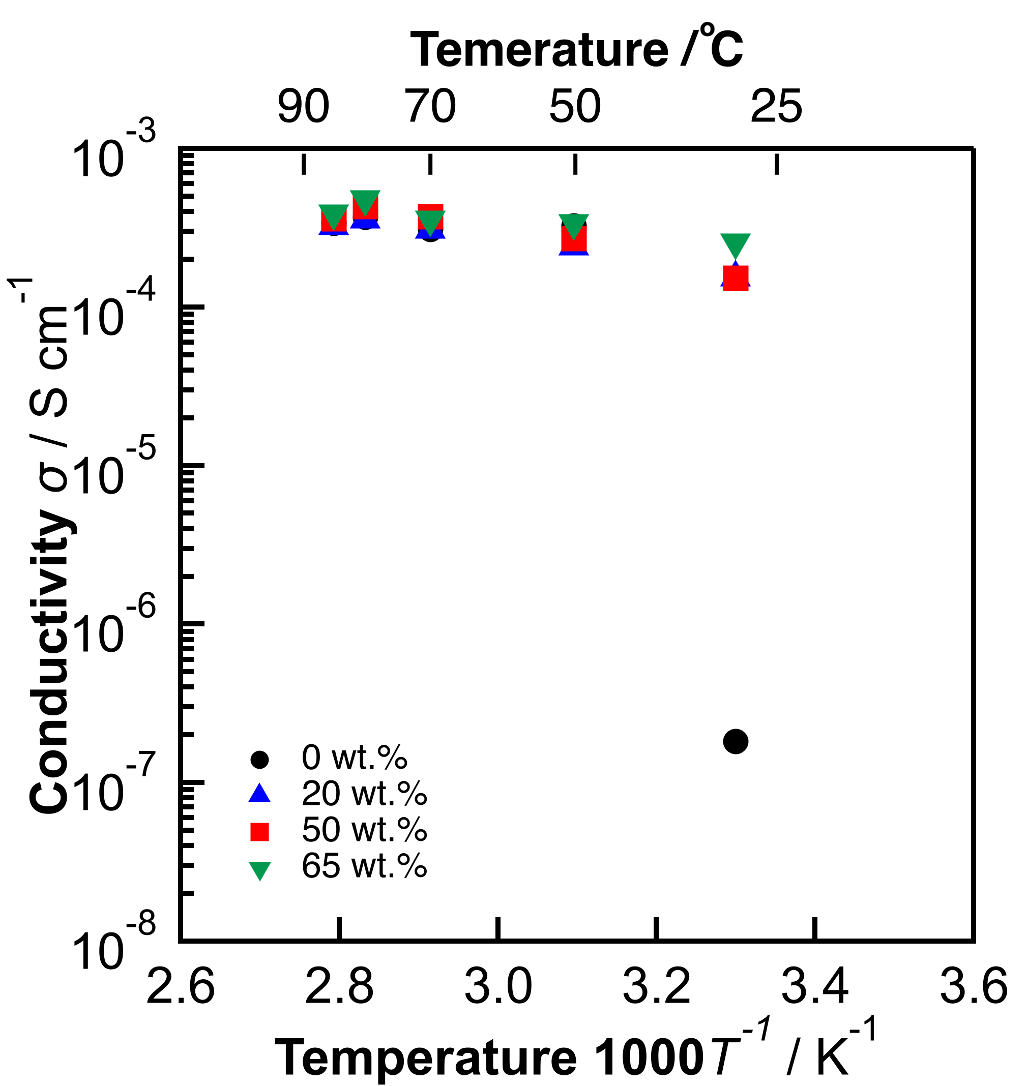


**Fig. S.4:** Proton conductivity of PVPA-coated W_18_O_49_ NWs (powder) at 80% relative humidity.

**Fig. S.5:** Mechanical strength of Pure PBI and PDDA 65%/W_18_O_49_/PBI membranes doped with PA (PADL = 8).

**Fig. S.6:** Images of PVPA 65%/W_18_O_49_/PBI membrane (left) showing agglomeration of NWs and PDDA 65%/W_18_O_49_/PBI membrane (right) showing its homogeneity.

**Table S.1:** Swelling ratio and water uptake percentage of the prepared membranes (PADL = 8).

|  | **Pure PBI** | **W_18_O_49_ / PBI** | **PVPA 65 % / W_18_O_49_ / PBI** | **PDDA 65 % / W_18_O_49_ / PBI** |
| --- | --- | --- | --- | --- |
| **Swelling ratio / %** | 140.9 | 137.77 | 176.84 | 206.48 |
| **Water uptake / %** | 52.7 | 113.48 | 85.6 | 88.9 |

**Table S.2:** Comparison of fuel cell performance exhibited by PBI-based different membranes.

| No. | Membrane structure | Current density (A/Cm^2^) | Power density  (mW/cm^2^) | PADL | Reference | Temp. |
| --- | --- | --- | --- | --- | --- | --- |
| 1 | PDDA/W_18_O_49_/PBI | 1.2 | 215 | 8 | This study | 150^o^C / 24 h |
| 2 | PBI/rGO-3 composite | 1.6 | 297 | 8 | [1] | 150 ^o^C / 24 h |
| 3 | PA-PBI (O-3) membrane | 0.2 | 144 | 11 | [2] | 160 ^o^C |
| 4 | Pyridine-bridged-oxypolybenzimidazole (PyOPBI) cross-linked brominated polyphenylene oxide (BrPPO) – P3 membrane | 0.28 | 84.6 | 14.2 | [3] | 160 ^o^C |

1. Maegawa, K., et al., *Development of polybenzimidazole modification with open-edges/porous-reduced graphene oxide composite membranes for excellent stability and improved PEM fuel cell performance.* Materials Chemistry and Physics, 2023. **294**: p. 126994.

2. Li, X., et al., *Dimensionally-stable phosphoric acid–doped polybenzimidazoles for high-temperature proton exchange membrane fuel cells.* Journal of Power Sources, 2016. **336**: p. 391-400.

3. Harilal, et al., *Cross-Linked Polybenzimidazole Membrane for PEM Fuel Cells.* ACS Applied Polymer Materials, 2020. **2**(8): p. 3161-3170.
